# Advances in Low-Dimensional Materials: Synthesis, Characterization and Device Application, 2nd Edition

**DOI:** 10.3390/mi17040421

**Published:** 2026-03-30

**Authors:** Tianyi Zhang, Xiaotian Zhang

**Affiliations:** 1 Department of Electrical Engineering and Computer Science, Syracuse University, Syracuse, NY 13244, USA; 2 School of Mechanical Engineering, Shanghai Jiao Tong University, Shanghai 200240, China; 3 Suzhou Laboratory, Suzhou 215123, China

In the past two decades, low-dimensional materials with fundamentally new physicochemical properties originating from quantum confinement effects have garnered significant research attention [1,2]. Their diverse morphology, chemical composition, and crystallographic phase, along with the ability to integrate different low-dimensional materials into van der Waals (vdW) heterostructures [3,4], have enabled the precise design and engineering of their functional properties, leading to a wide range of application domains such as computing [5], sensing [6], energy harvesting [7], catalysis [8], and quantum information science [9]. Recent efforts have been directed toward scaling up their synthesis [10,11], developing effective characterization approaches [12], and exploring device applications and monolithic integration [13]. With this field moving forward, it is increasingly recognized that the synergy among computational modeling, synthesis, interfacial engineering, characterization, and device research is essential to translate laboratory-scale discoveries into real-world applications.

The 2nd edition of the Special Issue “Advances in Low-Dimensional Materials: Synthesis, Characterization and Device Application” aims to bring these aspects together and advance the fascinating field of low-dimensional materials. It consists of five original research articles that highlight the latest developments in the field, from two-dimensional (2D) material-based composite film synthesis and high-throughput 2D materials screening, to functional applications in chemical sensing, biosensing, and energy storage. These articles provide a good overview of the field and share the common aim of bridging laboratory research on low-dimensional materials with scalable and practical applications.

The synthesis of 2D materials and their composites is a prerequisite for device demonstrations. In this Special Issue, Guerrero-Bermea et al. fundamentally investigated the synthesis of polyaniline (PANI)-graphene oxide (GO) composites through enzymatic polymerization of PANI in aqueous GO dispersions, and examined how the size of GO sheets (GO versus nanosized GO (nGO) prepared by prolonged sonication) affects the composite synthesis process [14]. A comprehensive set of characterizations were carried out to reveal the structural and chemical properties of the PANI-GO/nGO composites. It was found that PANI conformally deposits onto both GO and nGO sheets regardless of their size, and the interaction was governed by the vdW force between oxygen-based functional groups of GO and the secondary amino bond (-NH-) of PANI. Moreover, PANI-GO composites could be deposited onto gold surfaces, and molecular dynamic (MD) simulations shed light on the PANI-GO deposition mechanism at the nanoscale. This work provides insights into the design and synthesis of PANI-GO composites, which are directly relevant to device applications, such as the sensors examined in another article in this Special Issue [15].

Rapid, high-fidelity characterization of 2D materials is challenging yet crucial for efficient synthesis optimization and for screening suitable materials for device applications. Gasbarro et al. tackled the bottleneck of low-throughput analysis in the mechanical exfoliation of 2D materials by developing an open-source, GPU-accelerated software platform [16]. This platform enables rapid optical microscopy characterization and quantification of flake coverage and thicknesses for mechanically exfoliated 2D materials on various substrates. Notably, this platform is broadly applicable to a wide range of 2D materials including graphene, MoS_2_, MoSe_2_, WS_2_, WSe_2_, hexagonal boron nitride (hBN), etc. Compared to the existing open-source segmentation model, it could process over 200× more pixel data with a 60× reduction in processing time. This platform provides a valuable infrastructure for high-throughput analysis of both top-down exfoliation and bottom-up synthesis outcomes for 2D materials, facilitating yield and quality optimization in 2D materials development.

Building on the synthesis and characterization advances discussed above, the remaining three articles demonstrate the functional applications of low-dimensional materials and their composites. In the field of electrochemical sensing, Gohar et al. fabricated rGO/PANI composite films for pH sensing, a materials system closely related to that studied by Guerrero-Bermea et al., but fabricated with a distinct, scalable approach involving the dip-coating and subsequent photolithography patterning of rGO/PANI on flexible polyethylene terephthalate (PET) [15]. The patterned interdigitated electrode (IDE) arrays of rGO/PANI simultaneously serve as the pH-active medium and the electrical interface, enabling electrochemical sensors with simplified device stacks and reduced fabrication costs. The most precise measurement in the pH range of 2–10 was achieved with an optimized 80:20 rGO/PANI composition. The scalable composite IDEs reported in this work are useful for low-cost, mechanically flexible microsensors for field and in-line monitoring applications.

The biosensing application of low-dimensional materials was explored by Lee et al.; they developed a nanoscale biosensor based on carbon quantum dots (CQDs) for quantifying hemoglobin oxygen (HbO_2_) saturation [17]. The CQDs were synthesized from cellulosic paper by a facile one-step thermal treatment, and their fluorescence signal could be quenched through photoinduced electron transfer when interacting with HbO_2_. The peak fluorescence intensity of CQDs shows a strong linear correlation with HbO_2_ saturation, enabling the quantification of HbO_2_ saturation level and the results agree well with conventional spectrophotometric analysis. This work highlights the potential of CQD-based biosensors for clinical use across various physiological and pathological conditions.

Two-dimensional material-based composites are also emerging candidates for energy storage. Raza et al. designed a Co_3_O_4_@Ti_3_C_2_T_x_ MXene hybrid as a high-rate charge-storage electrode, leveraging the excellent pseudocapacitive properties of Co_3_O_4_ and the high electrical conductivity and mechanical flexibility of MXenes, while mitigating the intrinsic structural limitations of each single-phase material [18]. Structural characterization of Co_3_O_4_@Ti_3_C_2_T_x_ confirmed the uniform anchoring of Co_3_O_4_ nanoparticles onto MXene, with good structural integrity of both components. Electrochemical characterization revealed a dominant pseudocapacitive charge-storage contribution, reduced charge-transfer resistance, high specific capacitance (2379 F g^−1^ at 1 A g^−1^), and excellent rate retention in the Co_3_O_4_@Ti_3_C_2_T_x_ hybrid electrode, surpassing the performances of pristine Co_3_O_4_ and MXene electrodes. These results establish Co_3_O_4_@MXene as a robust platform for high-power supercapacitors. 

We hope this Special Issue serves as a useful reference for researchers who work in the field of low-dimensional nanomaterials and devices, and those who pursue interdisciplinary research in which low-dimensional materials could play an important role. As Guest Editors, we are grateful to the contributing authors for their timely and high-quality work, and to the reviewers who further strengthened the manuscripts with their expertise. Looking ahead, more synergies among the synthesis, characterization, and device applications are expected to accelerate the discovery of new materials and their heterostructures with novel physicochemical properties, and unlock the device applications of these materials beyond the capabilities of existing three-dimensional (3D) material systems. Moreover, as exemplified by the contribution of Gasbarro et al. [16], the growing integration of AI into materials research is also expected to play an increasingly significant role in advancing studies on low-dimensional materials.
